# Informality, violence, and disaster risks: Coproducing inclusive early warning and response systems in urban informal settlements in Honduras

**DOI:** 10.3389/fclim.2022.937244

**Published:** 2022-08-30

**Authors:** Laura E. R. Peters, Aaron Clark-Ginsberg, Bernard McCaul, Gabriela Cáceres, Ana Luisa Nuñez, Jay Balagna, Alejandra López, Sonny S. Patel, Ronak B. Patel, Jamon Van Den Hoek

**Affiliations:** 1 Institute for Risk and Disaster Reduction, and Institute for Global Health, University College London, London, United Kingdom; 2 School of International Service, American University, Washington, DC, United States; 3 College of Earth, Ocean, and Atmospheric Sciences, Oregon State University, Corvallis, OR, United States; 4 RAND Corporation, Santa Monica, CA, United States; 5 Pardee RAND Graduate School, Santa Monica, CA, United States; 6 GOAL Global, Dublin, Ireland; 7 Harvard Humanitarian Initiative, Cambridge, MA, United States; 8 Transcultural Conflict and Violence Initiative, Georgia State University, Atlanta, GA, United States; 9 Department of Emergency Medicine, Brigham and Women’s Hospital, Harvard Medical School, Boston, MA, United States

**Keywords:** coproduction, early warning systems (EWS), territorial gang violence, vulnerability, fragile and conflict affected contexts (FCAC), urban informal settlements, disaster risk reduction (DRR)

## Abstract

Anticipatory disaster risk reduction (DRR) is an essential human right for the ~1 billion people living in informal settlements who are disproportionately exposed to climate-related hazards due to their high vulnerability. Participatory approaches are recognized as being critical for effective and sustainable disaster prevention, mitigation, and preparation through to response, but research on how to coproduce anticipatory DRR with people living and working in informal settlements is scant. Their exclusion is even more pronounced in challenging contexts, such as those characterized by social-political fragility and violence. As a result, a significant portion of the global population is left behind in best practices tied to global DRR ambitions, with DRR actions working neither with nor for the people most at risk. The signal case of urban informal settlements controlled by territorial gangs in Tegucigalpa, Honduras, illustrates the need for new thinking on how to inclusively mitigate, prepare for, and respond to natural hazard-related disasters. Our research examines the coproduction of early warning systems linked with response capacities for floods and landslides through the case study of the international NGO GOAL’s work across the city with a focus on nine urban informal settlements with high levels of territorial gang violence. We explore how GOAL navigated informality and violent conflict to support the early warning and response system as an inclusive social process rather than a technical exercise. We identify four cross-cutting strategies employed by GOAL in support of local vulnerability reduction and capacity building based on a local systems approach. This research breaks new ground in identifying how to bridge the gap between knowledge and action in designing inclusive and sustainable early warning and response systems together with the millions of people around the world affected by the intersection of informality, violence, and disaster risks.

## Introduction

Anticipatory disaster risk reduction (DRR) is underrealized for the ~1 billion people living in informal settlements around the world who are disproportionately exposed to climate-related hazards and experience inequitable disaster impacts due to their vulnerability (UN Habitat, 2020; Dodman et al., 2022). The coproduction of anticipatory DRR—working alongside affected communities as partners in disaster prevention, mitigation, and preparation—is essential to translate multiple streams of information into early, sustained, and effective disaster-related actions (Carter et al., 2020). Best practices for “all-of-society engagement and partnership” (UNISDR, 2015, p. 13) must extend to those at the highest risk of disasters, but research on how to effectively coproduce anticipatory DRR in informal settlements—especially those characterized by violence and fragility—is scant.

The signal case of urban informal settlements controlled by territorial gangs in Tegucigalpa, Honduras, illustrates the need for new thinking on how to mitigate, prepare for, and respond to natural hazard-related disasters in urban fragile and conflict-affected contexts (FCAC). Fragility and conflict create and compound disaster risks, and impede development and humanitarian aid (Bangerter, 2010; Peters et al., 2019b). Where they are conducted at all, DRR actions in FCAC often remain limited and conform to reactionary, top-down, and technocratic approaches. In failing to include affected people throughout the process, such approaches risk being ineffective or even harmful, with the potential to entrench cycles of dependency and exacerbate interconnected risks for those facing the most risk to begin with (Clark-Ginsberg, 2021; Peters et al., 2021).

This article examines how the humanitarian international NGO GOAL coproduced an essential form of anticipatory DRR—early warning and response systems (EWRS) for landslides and floods—in nine urban informal settlements with high levels of territorial gang violence in Tegucigalpa. We begin with reviewing the literature on disaster risk creation and reduction in urban FCAC. We explain our materials and methods, describing the context of Tegucigalpa and GOAL’s programming, and how we developed the present research together with GOAL. We then present our results, discussing how GOAL coproduced the EWRS by leveraging a local systems approach. We discuss the difficulties and opportunities for engaging in places characterized by informality and territorial gang violence, and highlight tensions and challenges that remain for pursuing effective and sustainable DRR for all. We conclude with recommendations for researchers, practitioners, and policymakers on ways forward for coproducing EWRS in urban FCAC.

## Developing a conceptual foundation for inclusive disaster risk reduction as a human right

### Informal settlements face disproportionately high disaster risks

Disaster risks, including those related to climate hazards, are intensified for those living in informal settlements due to their heightened physical and social vulnerabilities, including increased exposure to hazards and inadequate access to support services (Revi et al., 2014). Informal settlements are defined as:

“Residential areas where (1) inhabitants have no security of tenure vis-à-vis the land or dwellings they inhabit, with modalities ranging from squatting to informal rental housing, (2) the neighborhoods usually lack, or are cut off from, basic services and city infrastructure and (3) the housing may not comply with current planning and building regulations, and is often situated in geographically and environmentally hazardous areas”

(UN Habitat, 2015, p. 1).

Informal settlements are settled and expand over time in unplanned ways through “unassisted self-help” (Napier and Rubin, 2002, p.8) often in hazardous areas in urban peripheries that have not been pursued for formal development (Napier and Rubin, 2002; Ajibade and McBean, 2014; Melore and Nel, 2020). Those living in informal settlements are often exposed to high levels of environmental degradation and risk, including in floodplains and steep or unstable slopes (Doberstein and Stager, 2013), where the cascading effects of climate-related hazards are even more pronounced, especially in the absence of risk-reducing and protective infrastructure (e.g., heavy rains contributing to flooding and/or landslides in part due to absent or dysfunctional stormwater pipes and flood control dams).

Hazards-based DRR solutions have emphasized eliminating informal settlements, empowered through the language of legality and criminalization where people occupy land over which they “have no legal claim, or which they occupy illegally” (UNHCR, 2014, p. 16). Also termed “slum clearance,” forcible eviction policies involve the relocation of people to ostensibly safer but often more isolated locations and forcing their adoption of state-owned housing, which perpetuates land and housing tenure insecurity (Morin et al., 2016). Such actions are increasingly recognized by the global community as failing to reduce disaster risks and in many cases leading to worse outcomes with a much higher social and financial cost (UNGA, 2018). These blunt tactics incur trauma and disruption on already highly vulnerable populations living in informal settlements (Ajibade and McBean, 2014) and fail to address the root causes of disaster (Morin et al., 2016) as well as their informality. They exacerbate the growth of informal settlements where there are not safer and appropriate alternatives (Napier and Rubin, 2002), sever trust in the government (Melore and Nel, 2020), and undermine community motivations to make environmental improvements (Ajibade and McBean, 2014). The chronic lack of investment in informal settlements perversely contributes to disaster risks while also impeding the potential for informal settlements to formalize through the gradual accumulation of land rights over time or official legal actions (Fernandes, 2011), effectively entrapping people in vulnerability.

More inclusive approaches to risk reduction in informal settlements are crucial from a human rights perspective. Regardless of legality, informal settlements are fundamentally human settlements (Zárate, 2016), and the inhabitants have a legitimate claim to international human rights. The right to DRR specifically has been made including in the Universal Declaration of Human Rights (UN, 1948) (Kent, 2001), and the International Covenant on Civil and Political Rights (UN, 1966) obliges states to ensure the ability for citizens, including those with tenuous housing and living in informal settlements without property ownership, to exercise their rights equally (UN, 1966). Shifting toward a rights-based approach “… understands informality as resulting from systemic exclusion and advances a set of recommendations for supporting and enabling residents to become full participants in upgrading” (UNGA, 2018, p.2) including the right to participate in actions taken over development and environmental concerns. A rights-based approach to DRR does not encourage people to reside in hazardous locations, even while it does not force them to leave places still deemed habitable. Rather, it recognizes that people live in informal settlements due to lack of better options and opportunities and therefore supports processes and actions that align with their unique needs and goals and contribute to their dignity.

### Fragility and violent conflict create and compound disaster risks

Increased exposure to hazards is both caused and magnified by the vulnerability forced upon people living in informal settlements. Academic research as well as global policy and practice agree that disasters are socially created and not natural (Ball, 1975; O’Keefe, 1976; Tiranti, 1977) even when they may be influenced by climate-related hazards (Raju et al., 2022). This means that climate-related factors, including climate change, should not be overstated as a single-factor cause of disasters, and that root causes—namely vulnerability—are at the core of disaster risks (Kelman, 2015; Kelman et al., 2016). Vulnerability encompasses “the characteristics of a person or group and their situation that influence their capacity to anticipate, cope with, resist and recover from the impact of a natural hazard (an extreme natural event or process)” (Wisner et al., 2004, p.11). Vulnerability is better understood as a socially created *process* rather than an inherent characteristic or status (Pelling, 2003) affecting people differently and manifesting in particular places, points in time, and situations (Tierney, 2019). Vulnerability results from people lacking access to and ownership of assets and resources over time (Wisner et al., 2004), which in informal settlements may take an extreme form of “persistent neglect and underdevelopment” (Gaisie et al., 2021, p. 2). Repeated disasters can contribute to the ever-increasing vulnerability or “ratchet effect” (Chambers, 1989) of affected populations.

Vulnerability explains why even the same climate-related hazards do not wield uniform effects across social groups, with the most marginalized who are continuously held in risk being the most frequently and severely impacted by disasters (Hilhorst and Bankoff, 2022; IPCC, 2022). It is thus necessary to engage with social and political conditions and processes in order to understand and act inclusively upon disaster risks (Kelman, 2020). Yet, this is impeded by the deliberate depoliticization of disasters (Siddiqi, 2018) including in areas affected by fragility—where the state is unable or unwilling to apply authority and does not provide appropriate basic services to the population—and conflict—the use of armed force between parties. Altogether, such areas are known as fragile and conflict-affected contexts (FCAC).

**FIGURE 1:**
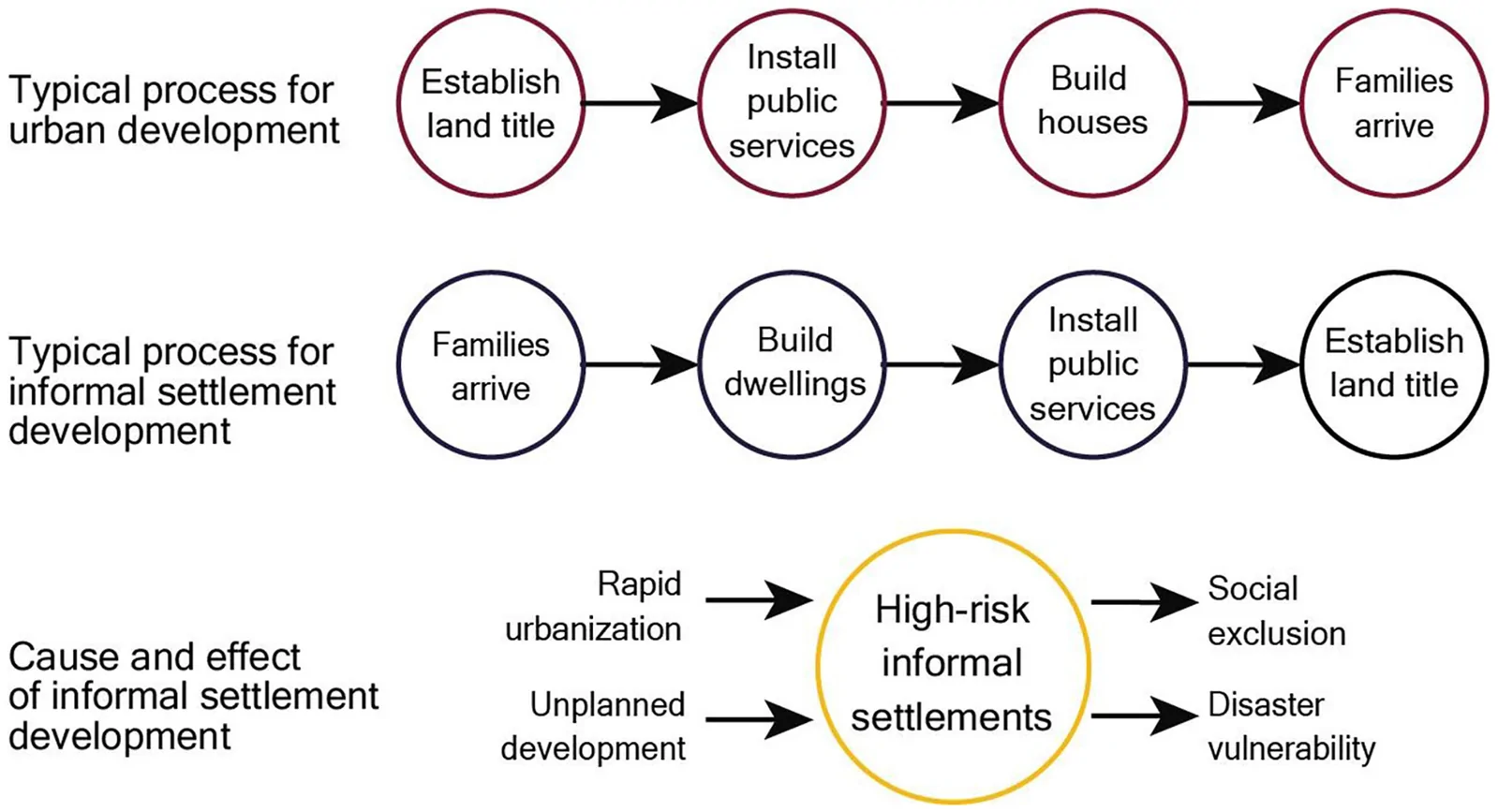
Conceptual process for the contributions of informal settlement development to disaster vulnerability. Adapted from GOAL’s Resilient and Inclusive Neighborhood Approach (RINA) (2022).

Urban informal settlements with territorial gangs in Tegucigalpa and other locations around the world may experience fragility (*via* informality) and conflict (*via* gang violence). Informality is an expression of fragility, where state institutions focus on policing and security within an eroded authority and control, but are notably lacking in providing reasonable infrastructure and public services. People living in informal settlements are kept at the social, political, and geographic peripheries due to formal governance being unable and/or unwilling to provide for the population’s basic needs. In some cases, the lack of integration can be traced to a central government’s inability to sufficiently expand services during rapid and unplanned urbanization to the physical peripheries where informal settlements tend to be located (Morin et al., 2016) (see Figure 1). In many cases, however, the government denies people who live in informal settlements basic resources, services, and rights, which can even be codified through law where the state criminalizes informal settlements. The lack of integration with urban services is exacerbated by insecure land and housing tenure (Ajibade and McBean, 2014; Usamah et al., 2014), and people may have no choice but to construct substandard or temporary housing due to a combination of poverty and not being allowed to construct something more permanent in such places (Morin et al., 2016). The fissure between populations living in informal settlements and the formal government produces a governance void that enables other actors to take control and exert their own authority.

Violent social conflict can manifest through territorial gangs, which are “midway between criminal groups and community groups: they try to gain control of a territory to oversee all criminal activities in that area and/or to ‘protect’ the people living there” (Bangerter, 2010, p. 391). Concentrated in disadvantaged and marginalized urban areas worldwide, territorial gangs can offer an alternative to an absent or hostile state and rally communities under the flag of social justice and protection (Bangerter, 2010)—however violent it may be in its execution—and the long-term presence of territorial gangs enables them to establish authority and enforce their rules as law. Territorial gangs exert violent control over neighborhoods and populations and conduct warfare with feuding gangs and other state and non-state entities intruding in their territories or interfering with their activities and agendas. Territorial gangs do not typically seek to overthrow the state or challenge its authority (Bangerter, 2010) and often criminally collude with state actors even while they may also clash, which contributes to persistent violence, fragility, and a culture of impunity (Auyero and Sobering, 2019).

Territorial gangs both reflect and create enormous humanitarian and development needs and challenges: “poverty and marginalization are at one and the same time the causes and the consequences of gangs” (Bangerter, 2010, p. 392). In urban informal settlements controlled by territorial gangs, the government creates vulnerability through their exclusionary treatment of informal settlements, and then territorial gangs, which thrive in vulnerable and marginalized contexts, create further vulnerability through organized violent crime. A lack of formal services may leave needs unmet generally as well as specifically related to disasters. City and state governance often excludes those living in informal settlements from disaster-related activities and decision-making (Alvarez and Cardenas, 2019; Clark-Ginsberg, 2021). Those residing in informal settlements may not even be accounted for in disaster preparation through to relief and response (Morin et al., 2016) nor in broader sustainable development monitoring (Van Den Hoek et al., 2021). As a result, those living in informal settlements may rely solely on NGOs for services (Miles et al., 2012) that may demonstrate a poor understanding of their needs, and the most violent and insecure contexts often lack even these outside interventions (Peters, 2021).

### Coproducing inclusive early warning and response systems

A central challenge in urban informal settlements controlled by territorial gangs—and FCAC more broadly—is in finding ways to conduct anticipatory DRR rather than resorting to purely reactionary forms of humanitarian support deployed only after disasters occur. One type of anticipatory DRR includes early warning systems (EWS), which are defined as:

“An integrated system of hazard monitoring, forecasting and prediction, disaster risk assessment, communication and preparedness activities systems and processes that enables individuals, communities, governments, businesses and others to take timely action to reduce disaster risks in advance of hazardous events”

(UN, 2016, 17/41).

EWS for climate-related hazards depend on climate services and the ability to predict and detect potential hazards, since timely climate-related information can enable people and institutions to improve ex-ante decision-making (Tall, 2013) and implement predetermined actions to protect people from disaster impacts (Kumar, 2022). However, strengthening technical capacities and communication mechanisms alone is not enough to prevent disasters, and EWS must engage with social factors and processes to be effective. As the above definition implies, EWS must connect knowledge with action, making the development of response systems an integral part of effectiveness. This article adopts the terminology of early warning and response systems (EWRS) to highlight the importance of cultivating the linkage between early warning and response.

Rather than EWRS merely being activated once a hazard is detected or anticipated, engaging the social process means that pre-hazard components (such as education, training, and collaboration) (Baudoin et al., 2016) and post-hazard components (such as setting up response systems) (Mountfield, 2014) are taken into account through long-term communication, discussion, and participation processes (Kelman and Glantz, 2014). By doing so, EWRS become more accessible, interpretable, and actionable by affected people and service providers that support them, including building capacity for early and effective action before a hazard is realized as a disaster. Integrating climate services with social factors and processes can help to close what is referred to as the “usability gap” in EWS (Dupar et al., 2021)—a euphemistic term, as such failures result in morbidity and mortality, economic losses, and destruction of critical assets.

A large body of research and practice affirms that it is necessary to inclusively engage with people and their lived experiences to achieve the effective, sustainable, and equitable reduction of disaster risks (Maskrey, 2011; Shaw, 2012) including in EWRS (Walker, 2013; Baudoin et al., 2016; Sufri et al., 2020). This best practice is reflected in global policy processes such as the Sendai Framework, which emphasizes that EWRS “should occur through a participatory process” (UNISDR, 2015, p. 21), as well as its predecessor, the Hyogo Framework, which recommends “people-centered” approaches to EWRS. Various forms of community participation under this broad banner of community-centric EWRS dovetail with a rights-based approach to informal settlement upgrading (UNGA, 2018). While some forms of community-based approaches conform more closely with top-down and neoliberalist agendas that normalize insecurity (Gladfelter, 2018), community-driven approaches ideally originate from and are co-led by the community itself and lead to improvements that support their collective wellbeing.

Coproduction, one community-driven approach, seeks to develop “solutions through legitimate processes that draw on diverse and credible expertise with, by and for those best placed to use them” (Chambers et al., 2021, p. 983) through a partnership between citizens and service providers (Parks et al., 1981). Instead of relying on top-down and technocratic interventions at the hands of “experts” (Hewitt, 1983), practitioners collaborate closely and over sustained periods with communities, giving them control over agenda setting, design, implementation, and management of programming. In doing so, they ideally ensure that EWRS align with community priorities and needs. The role of the practitioner is one of supportive partnership, which can take many shapes, including through developing relationships, connecting communities, providing support in advocacy, supporting the creation of knowledge and awareness, providing financial resources and technical expertise, assisting with problem solving, and providing platforms for knowledge creation and communication (Peters et al., 2021).

Creating an effective and sustainable EWRS is argued to be best done through coproduction (Marchezini et al., 2018; Izumi et al., 2019; McLennan, 2020). Coproducing EWRS enables more accurate and community-relevant information (Sufri et al., 2020) and more effective modes of communication (Hamidazada et al., 2019) leading to effective early action. The coproduction process recognizes that communities should not only be defined by their vulnerability but also their capacity and some level of agency in shaping their risk environment. Even informal settlements have features that can bolster EWRS, including: trust within the community, social cohesion, an attachment to place, high levels of community participation and investment, and regular communication and sharing of information (Usamah et al., 2014). People living in informal settlements can use their collective agency to reduce their disaster risks when they have substantive access to services and information (Miles et al., 2012; Gaisie et al., 2021) and are included in governance, planning, and management (Ajibade and McBean, 2014). Inclusive coproduction, especially penetrating into underrepresented and underserved social groups as well as informal and formal systems of governance, supports tailoring assistance to the varied needs, assets, and capacities within a community (Barbelet et al., 2021).

### The action-knowledge gap for coproducing EWRS in urban FCAC

Despite the increasing awareness of the benefits of coproducing EWRS, not much is known about how to coproduce EWRS—or create functioning EWRS through any approach—in urban FCAC. Despite the demonstrated benefits of inclusive approaches, flood management in informal settlements, for example, is not typically participatory since the residing populations are often seen as part of the problem (Amoako and Inkoom, 2018).

Counterproductive DRR solutions are seen where the government does not have the capacity or willingness to equitably reduce disaster risks (Peters et al., 2019b; Patel et al., 2021), with exclusionary actions often taken in FCAC. For example, in 2017, a fatal landslide in Mocoa, Colombia, disproportionately affected conflict-induced internally displaced persons (IDPs) who settled in dangerous locations on steep unstable slopes. This population was left out of disaster preparation and rehabilitation planning, with some government officials maintaining that they “had no rights since they ‘chose’ to live in a hazard-prone area,” and conformed to top-down approaches to DRR without community engagement (Siddiqi et al., 2019, p. 9). Likewise, in informal settlements in Lebanon, people face intersecting conflict and disaster vulnerabilities, yet they are not represented in policy, planning, and funding for DRR (Peters et al., 2019a). Closing the action-knowledge gap in coproducing EWRS in urban FCAC like informal settlements controlled by territorial gangs in Tegucigalpa has the potential to guide inclusive DRR for some of the most vulnerable and marginalized populations in some of the most challenging contexts around the world.

## Research methodology and case study background

### Research methodology

This research employed a collaborative process between academic researchers and practitioners at the humanitarian NGO GOAL to investigate GOAL’s development and implementation of EWRS in nine urban informal settlements controlled by territorial gangs in Tegucigalpa, Honduras (2013–2022). We began the research process by discussing the conceptual foundation for disaster risk reduction and creation described in Section 2, analyzing GOAL’s documents within this framing to develop an initial narrative of the case, and then initiating an iterative process of team discussion and writing.

We conducted a review of 58 published and internal documents in English and Spanish produced by GOAL between 2011 and 2021 describing how they coproduced the EWRS. This collection of documents includes technical preparatory reports from before the EWRS program was implemented, community census summaries, hazard maps of areas around Tegucigalpa, and synthesis reports. This review provided a baseline of information to inform the case study.

Following the document review and initial case development, we engaged in team discussions to better understand the document contents and contextualize them in terms of key learnings for urban FCAC. The aims of these conversations were three-fold: (1) to confirm the accuracy and representativeness of the initial case narrative, (2) to gain a more detailed understanding of the underlying reasons for engagement decisions and the impacts of these decisions on EWRS coproduction, and (3) to discuss tensions and challenges that remain around EWRS coproduction. Successive rounds of discussion between study authors including GOAL leadership took place *via* remote meetings and in a shared document to identify emergent themes and findings, and seek feedback and clarification. The study findings presented here represent the outcome of this iterative research process.

### Background on the case study

Honduras is one of the most vulnerable countries to natural-hazard related disasters within Central America (Suárez and Sánchez, 2012), repeatedly experiencing hurricanes, droughts, floods, and earthquakes. Hurricane Mitch in 1998 is considered by the United Nations to be the worst disaster in Latin America over the last 200 years, resulting in a loss of more than 20 years of social and economic development in the country (Suárez and Sánchez, 2012). Tegucigalpa was also severely affected by Tropical Storm Agatha (2010), which resulted in widespread flooding and landslides. Since 2020, ~1 in 4 Hondurans (2.8 million of the total 10.1 million population) has been in need of humanitarian aid, and 937,000 people were displaced due to disasters in 2020 alone (IRC, 2022).

These disasters occur in a vulnerability-laden context. Many residents lack regular access to basic services like potable water (44%) and sewage systems (53%) (BID NDF, 2016, p.16), 63.3% of households live in poverty with 37.3% in extreme poverty (INE, 2018), and approximately 80,000 families are estimated to be living in poor quality housing in disaster-prone areas. Tegucigalpa is made up of nearly 500 *barrios* or neighborhoods, and informal settlements make up 52% of the population and are home to much of the city’s growing population. Tegucigalpa also has a high level of violent crime with a homicide rate of 41 people per 100,000 people, making it one of the most violent cities in the world (Seguridad, 2020). This violence is largely perpetrated by territorial gangs including Mara Salvatrucha (MS-13) and the 18th Street Gang (Barrio 18) and command a high level of membership especially among young men (Bangerter, 2010; HRW, 2021).

GOAL’s work in Tegucigalpa is tied to the city’s disastrous history. Tegucigalpa (1,514 km^2^), Honduras’s capital city and home to approximately 1.3 million people (INE, 2018), has been heavily impacted by disasters. Tegucigalpa is situated in a narrow valley with steep slopes with the Choluteca River flowing north to south through the city, which frequently experiences major landslides and floods. The rainy season typically lasts from June through October, and floods are most common in the early months (April through June) when intense rainfall events lead to 30–60 mm of accumulated precipitation within one to one-and-a-half hours. These types of events contribute to the rapid overflow of small channels such as the La Orejona, Mololoa, Las Burras, La Seca, El Sapo, Grande del Norte, and El Cacao streams as well as pluvial flooding in streets. These events are also possible but less common during the later months of the rainy season. Another type of flooding can occur with extreme hydrometeorological events in August and November, such as tropical storms or hurricanes, which bring both long-lasting rainfall and rapid accumulations ranging from 60 to 150 mm per day. These types of events can contribute to problems of overflow in large channels such as the Chiquito, San José, and Choluteca Rivers.

Tegucigalpa also has a history of more dramatic flooding and landslides. Most influentially, ~900 mm of rain fell in a 4-day period from 27 to 31 October, 1998 during Hurricane Mitch (Hellin et al., 1999), and flooding along the banks of the Choluteca led to the city center, including lower-income neighborhoods, being inundated by up to 10m of water and largely destroyed the center of the city (ECLAC, 1999). The rains also triggered landslides, including a massive landslide (Bariche, weighing 6,000,000 m^3^) in the city which temporarily formed a dam and exacerbated flooding. The combination of flooding and landslides led to historical deaths, devastation, and damage. While this hurricane was a once-in-200-year event, it could happen again, especially since people still live in the same flood risk areas with increased risk levels. EWRS, which broadly did not exist in 1998, alongside vulnerability reduction are the primary defenses to prevent these events from unfolding so disastrously again. As a part of its public awareness programming, GOAL installed signs in buildings throughout Tegucigalpa to depict where flooding levels reached as a sobering reminder of what the future might look like in the absence of effective DRR.

**FIGURE 2:**
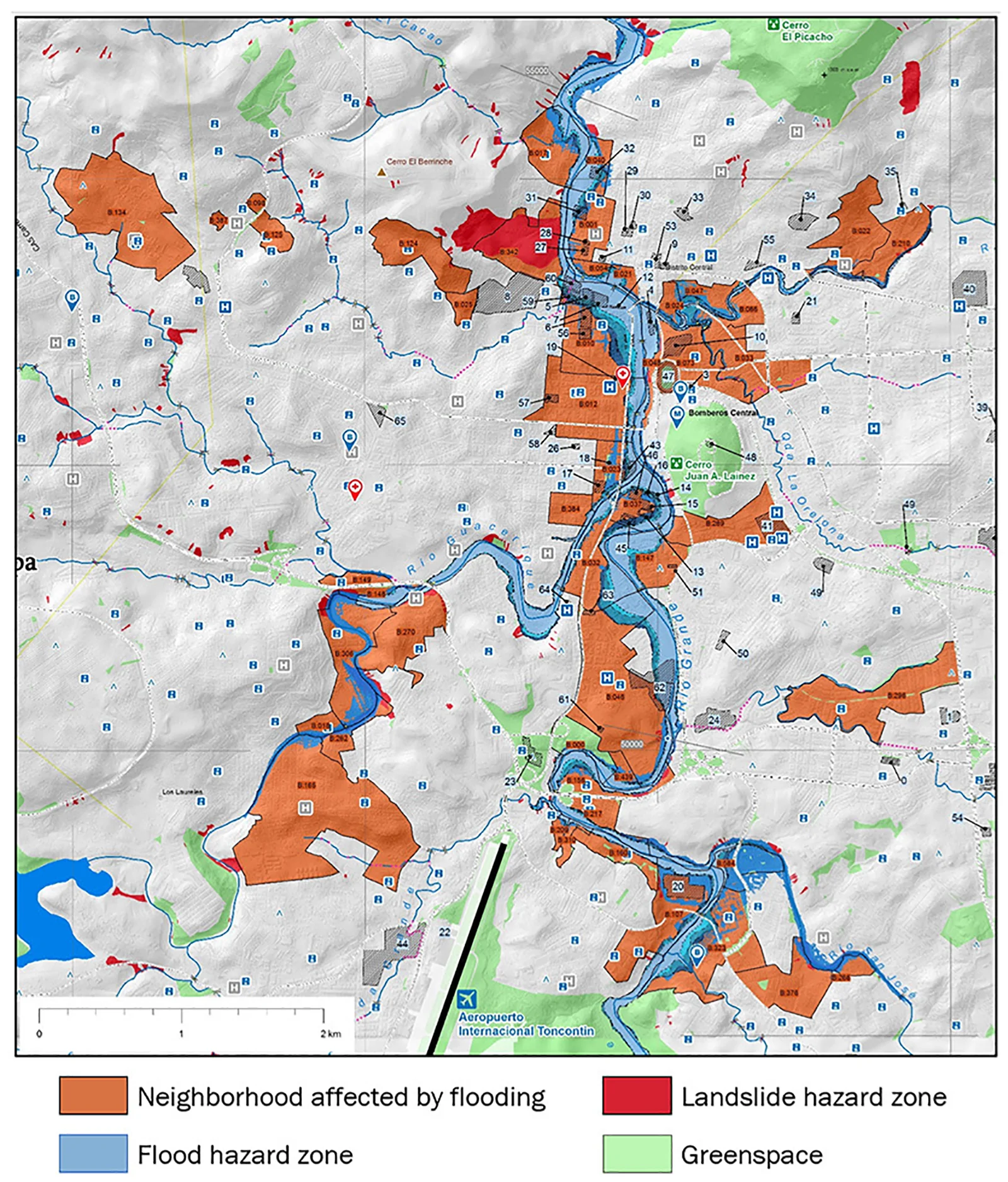
Flood hazard map for the Choluteca River in Tegucigalpa. Blue depicts flood hazards projected for different rainfall events and risk scenarios. Red depicts landslide hazards along the river that can exacerbate flooding. Amber depicts neighborhoods historically affected by and which must be prepared for flood hazards. Key local resources relevant to flood risk preparedness and response including hospitals, schools, and meteorological monitoring stations are also marked on the map. Credit: GOAL 2013.

**FIGURE 3:**
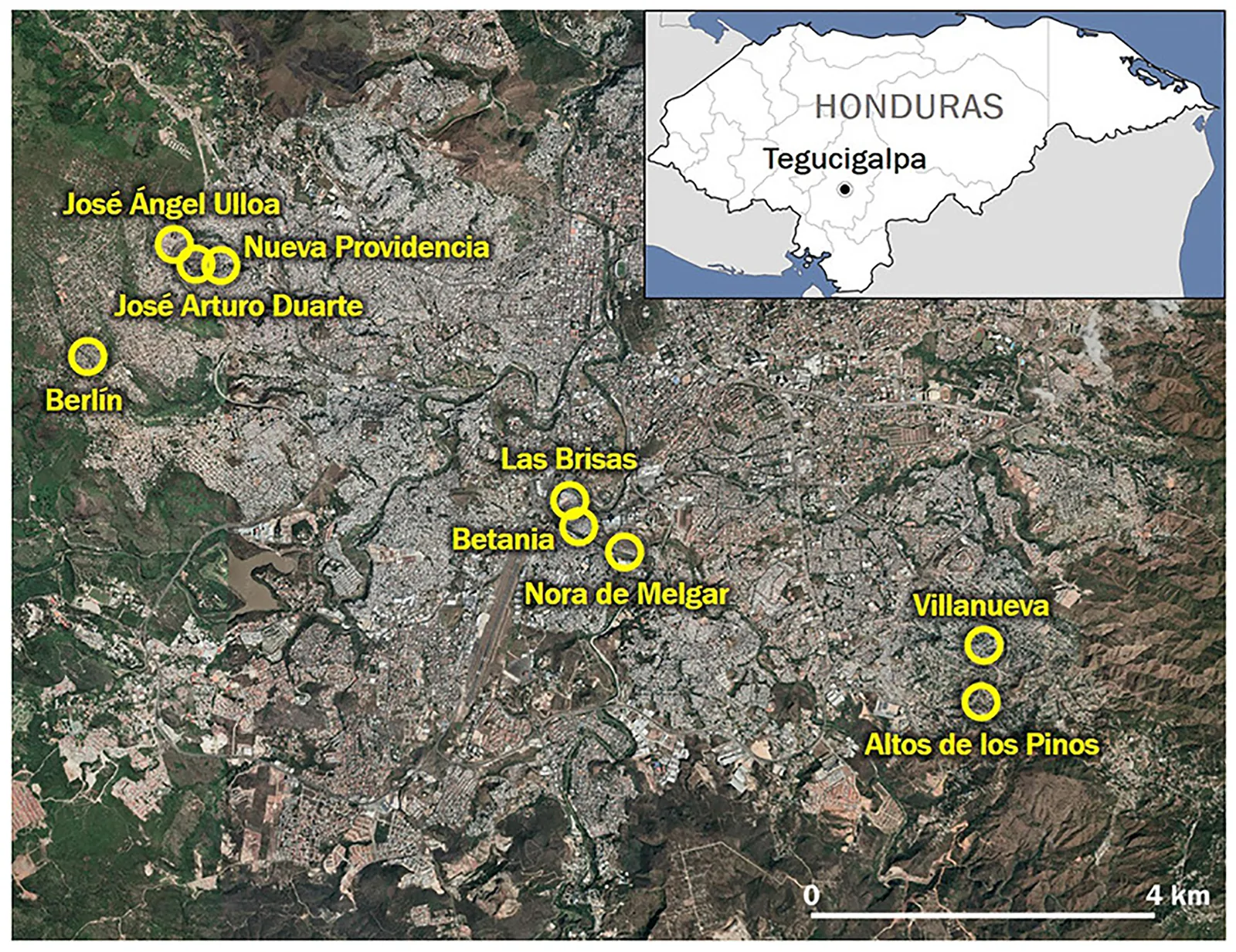
Map of nine study informal settlements (yellow circles), with background satellite image of Tegucigalpa, Honduras. Credit: Authors.

GOAL entered Honduras in 1998 in response to the enduring consequences of Hurricane Mitch, and the organization began urban programming in gang-controlled neighborhoods in Tegucigalpa in 2003. Damage from Tropical Storm Agatha alongside high-risk informal urbanization and a lack of warning and response systems motivated GOAL to begin working on flood and landslide EWRS in Tegucigalpa in 2010. GOAL launched its EWRS work by coproducing a series of flood and landslide risk maps based on historical flood levels and landslides, along with future risk scenarios applied along the entire 300 km catchment of the Rio Choluteca. This represented a massive participatory effort bringing together national and municipal authorities from multiple cities together with international agencies, technical advisors, and other representatives and stakeholders, and these maps (publically available at https://resiliencenexus.org/urban-resilience/ewrs/) and related EWRS Operation and Maintenance Manual now form the basis for the EWRS along the Choluteca River. GOAL has worked with nearly 40 high-risk neighborhoods since then, which are highlighted in amber in the flood hazard map (see Figure 2), with sustained engagement in nine exceptionally high-risk informal settlements (included in Figure 2 and outlined in Figure 3) located predominantly in the peripheries of the city and along the river floodplain.

A combination of factors results in high risks in these nine settlements. One is housing: over 70% of the houses in these nine settlements are self-constructed, and, in almost all cases, dwelling construction has been undertaken without any outside technical assistance or formal consideration of hazards. Services are inadequate and extend to surface water drainage problems contributing to flood and landslide risk due to increased levels of uncontrolled surface runoff, soil saturation, and erosion. Social problems are rife, including the high prevalence of gang violence. Underlying these challenges are deep structural problems related to social and economic exclusion. Households are extremely poor with an average monthly income of Lps 3,082 (134 USD), and approximately two-thirds of residents are employed in the informal economic sector composed entirely of small and micro businesses including *pulperías*, or small grocery stores, which play a significant role in the social fabric of informal settlements.

### Background on GOAL’s EWRS programming

GOAL coproduced EWRS in these settlements to prepare for and respond to small- and large-scale floods and landslides associated with high precipitation events in high-risk areas. The nine urban informal settlements feature conditions “typical” to many informal settlements in their exposure to an array of hazards and ongoing processes of vulnerability. We aggregated GOAL’s experiences coproducing EWRS in these nine settlements, because they used the same approach across these neighborhoods as well as through city-wide initiatives with similar results. The process followed a “first-mile approach,” which includes people in the development of agendas and plans through to implementation (Kelman and Glantz, 2014), and GOAL engaged with a wide range of stakeholders beyond traditional gatekeepers (i.e., working beyond those in established leadership positions) at different levels of formality.

The EWRS involves four components, which are conceptualized as ongoing and mutually overlapping social processes, rather than being linear and sequential stages:

1. *Conducting risk awareness*: GOAL supported risk awareness for communities, authorities, and other stakeholders. This process involved creating knowledge of the multiple factors influencing disaster risks, including the spatial and social distribution of hazards and vulnerabilities, and communicating these risks to the broader public, particularly first responders and communities and households at risk.
2. *Establishing and monitoring alert thresholds*: GOAL collaborated with communities and other stakeholders to establish flood and landslide alert thresholds and monitor appropriate meteorological data based on the coproduced risk knowledge. Different thresholds for evacuation were developed for different communities based on their risk levels, with specific community-level vulnerabilities and needs taken into consideration. A software system was also created to collate monitoring data and automatically determine community-level alerts in near-real time.
3. *Disseminating alerts to all relevant stakeholders*: GOAL coproduced alert dissemination protocols, encompassing the format and content of alerts, unique to each neighborhood and first responder agencies.
4. *Strengthening response capacity and contingency planning*: GOAL developed plans in conjunction with communities, municipalities, and relief agencies to enhance existing community-level capacities for responding to alerts and related contingency planning (see Table 1).

GOAL’s programming was found to be effective and sustainable (scoring 4.5 out of a possible 5.0) in an external assessment conducted by Florida International University (2018). Some of the strongest aspects of the EWRS programming’s effectiveness included people (including children) knowing the risks they live in, safe zones, and what to do in the case of an emergency through drills and training, and improved drainages and walls reducing the occurrence of floods and landslides. Some of the weakest aspects of its effectiveness included only 20% of the communities participating in the drills, and many people still living in areas of high risk with poorly marked evacuation routes. In terms of sustainability, some of the strongest aspects included people being empowered and proud of their community, and willing and able to share what they have learned with others as well as collaborate toward community improvement efforts. Some of the weakest aspects of its sustainability include community members expressing that GOAL is necessary for motivating the municipality to take certain DRR actions, especially in light of the lack of funds and other support for DRR. Table 2 presents data gathered on the program’s impacts on DRR and social cohesion derived from ~40 surveys from heads of households.

## Results

GOAL coproduced the EWRS through a local systems approach. Local systems entail “the amalgamation of formal and informal stakeholders that together provide the core sets of services that local populations rely on for their basic needs” with the acknowledgment that especially in FCAC “the state is not the sole provider of services” (Patel et al., 2021, p. 5). Since these services are foundational for effective and sustainable EWRS, GOAL’s programming explicitly sought to engage with and strengthen core local systems within the informal settlements. In order to do so, GOAL coproduced the EWRS not only with the people living and working in informal settlements but also with the formal and informal actors and systems of governance involved in the provision of services and protections and took into consideration the assets and resources they depend on to do so. We describe four strategies related to this local systems approach that GOAL employed in the following subsections (4.1–4.4).

### Identifying and empowering key local systems actors

GOAL sought to identify, engage, and empower key local systems actors. The central focus was not necessarily to coproduce the EWRS with established official or unofficial leaders or specific target groups of vulnerable populations, but to do so with local actors based on their actual or potential role within critical local systems. GOAL sought to empower these key local systems actors and increase their leadership capacities within the EWRS. GOAL also facilitated building partnerships between key local actors and other stakeholders including local government departments, academic institutions, private sector businesses, civil society organizations, and community leaders to encourage their capacities to co-develop and lead collaborative initiatives. This work was significant in the context of informality and territorial gang violence, because programming helped to empower those often victimized by violence as well as repair relationships strained by violence between key local actors and other stakeholders.

GOAL found that women and youth play central roles in local systems in the informal settlements, and GOAL engaged them as key actors within the EWRS. Women in particular are invested in their communities and are motivated to mobilize collective actions to reduce risks and improve conditions. Women own the majority of small businesses such as neighborhood stores, cafes, and *pulperías*. *Pulperías* are small convenience stores that also function as casual gathering places, and they arguably form the loci of social life, knowledge exchange, resource distribution, and relationship-building in informal settlements in Tegucigalpa. Similarly, youth are confronted with significant risks in informal settlements, which tend to make them highly motivated to support interventions that could result in a dignified future for them, their families and friends, and the wider community.

**FIGURE 4:**
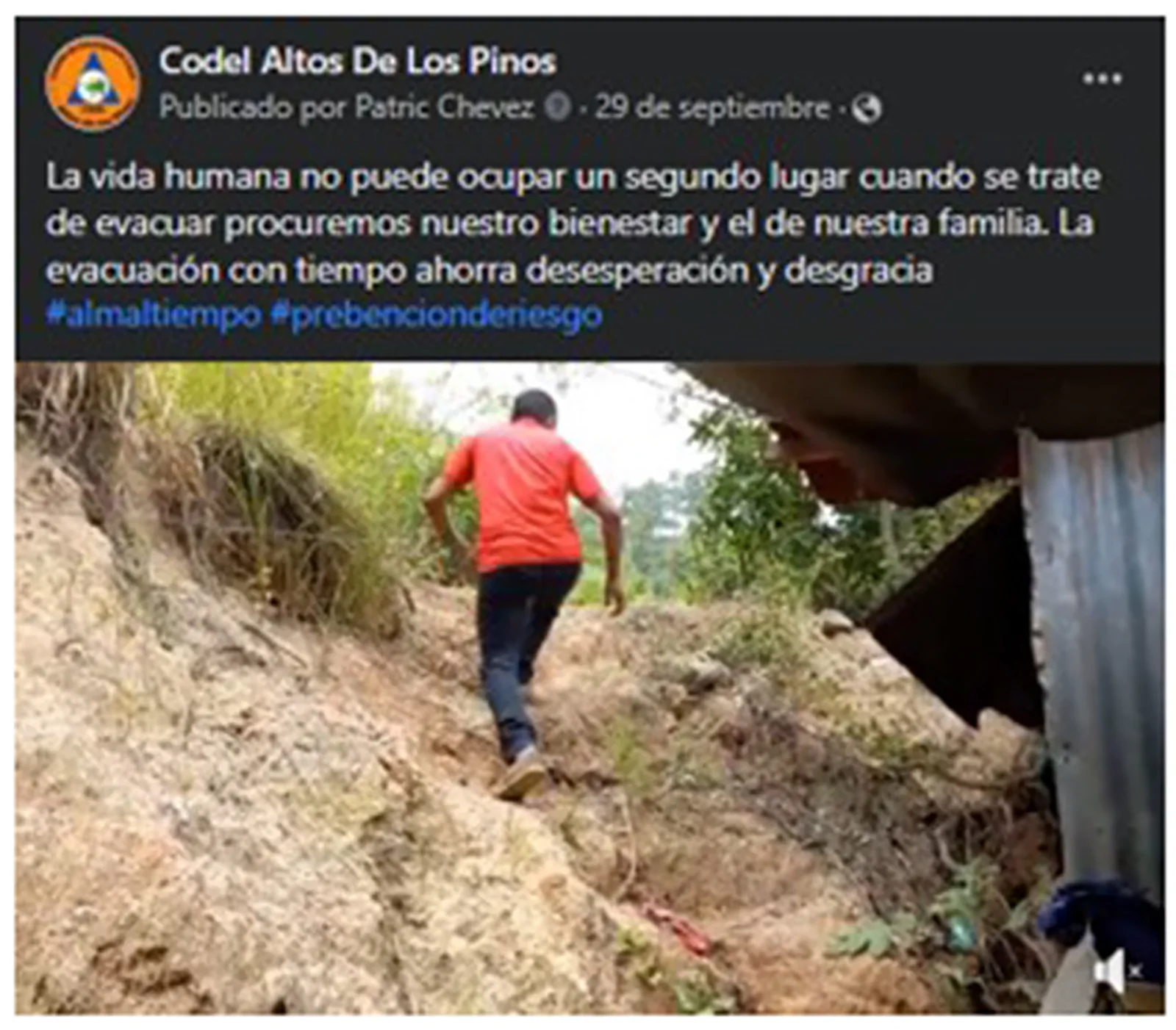
Risk communication in the Altos de los Pinos neighborhood. Translation: “Human life cannot take second place when it comes to evacuating. Let’s seek our wellbeing and that of our family. Timely evacuation saves despair and misfortune.” Credit: GOAL, 2021.

Women and youth leaders played key roles in developing risk knowledge, interpreting risk information, and galvanizing action. Based on their social positioning, women and youth had up-to-date knowledge of multiple hazards and risks, intersectional vulnerabilities, and “off-the-books” dynamics within their neighborhoods, which helped parse out specific needs of different groups and households. Their place-based knowledge made them well-suited to communicate tailored risk information with the most vulnerable (see Figure 4). Their social positioning also granted them influence; women and youth motivated other community members to participate in the coproduction process and build trust in a context where trust is hard to win and easy to lose. Ways they did this included facilitating participatory mapping exercises, conducting community visits, and organizing initial meetings and focus groups with other community leaders and members in ways that made people feel heard. In some cases, people were provided with small payments or meals to compensate them for their time, which signaled that their participation in the EWRS was valued.

**FIGURE 5:**
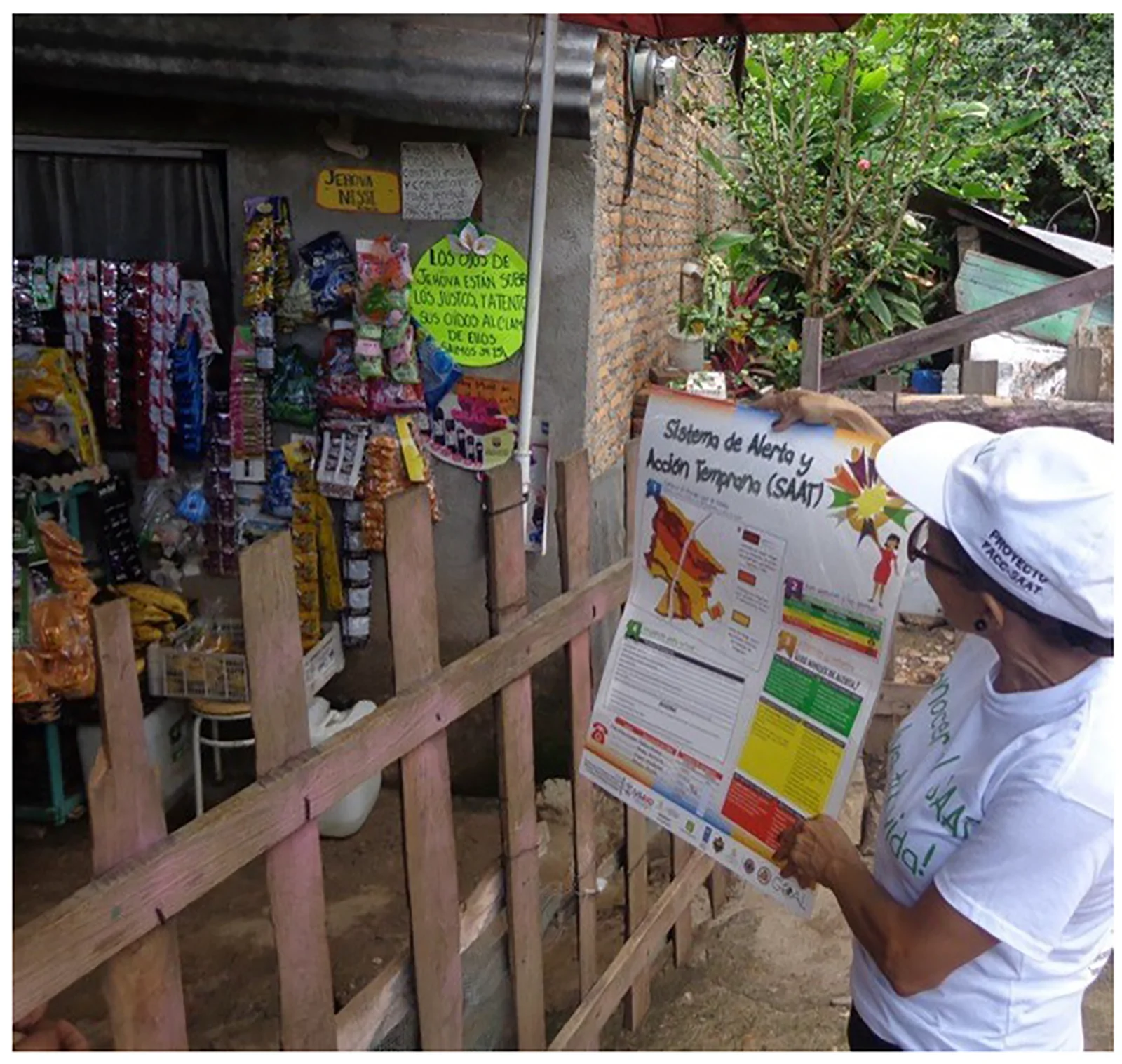
A “prevention preacher” makes house-to-house visits to raise awareness and knowledge about the EWRS. Credit: GOAL, 2016.

Despite being in a context where trust can be undermined by violence and conflict, women and youth were able to communicate risk information in ways that helped lead to changes in public beliefs, behaviors, and practices, and strengthened the ties between knowledge and action. The women and youth GOAL worked with leveraged their trust and influence to disseminate information, updates, and warnings quickly and across appropriate channels. Format and content of alerts were crucial; women and youth used a variety of channels—including email, mobile phone SMS messaging, VHF radio, word of mouth, broadcast media, social media, megaphones, and home visits—to reach all households, including those most at risk, in line with the city and national authority alert dissemination. Commonly known systems, such as siren blasts already used by local firefighters, were used whenever possible to ensure recipients easily understood warnings. Some also volunteered as “prevention preachers” to disseminate hazard maps and educate the community on what they should do in response to specific alerts through door-to-door visits (see Figure 5) as well as public presentations in bus stations, *pulperías*, and other public places.

**FIGURE 6:**
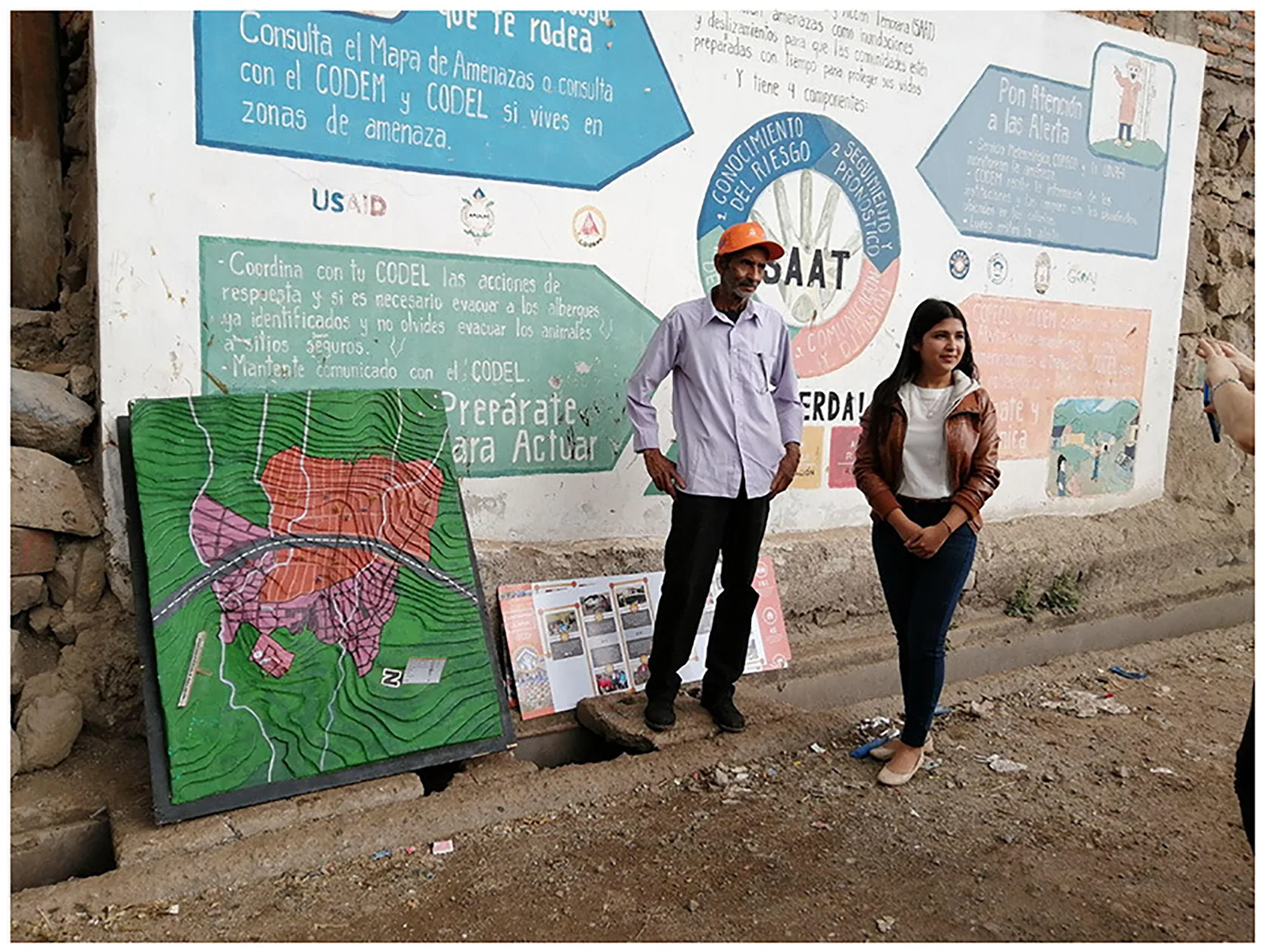
Community youth painted a mural illustrating the four key components of the EWRS—Know the risk that surrounds you. Pay attention to alerts. Get informed and communicate. Get ready to act.—along with a three-dimensional hazard map of the neighborhood. Credit: GOAL, 2020.

Youth participated in social cohesion programming and played a significant role in both preparation and response capacities through the establishment of youth groups trained in disaster preparedness and community awareness campaigns. For example, GOAL supported communication and risk awareness through the use of comedy and art—strategies codeveloped with youth to engage their peers and the broader community. “Disaster risk management weeks” were held in neighborhood schools around the International Day for Disaster Risk Reduction, and recreational activities like games, painting competitions, and the development and use of scale community models were used to increase awareness and knowledge of risk among children. Youth also created public murals through an arts program to increase awareness about EWRS (see Figure 6). Youth leaders were trained and also assisted with early evacuation for those with high vulnerability, including the elderly and those living with special needs, and they participated in youth local emergency committees and brigades for search and rescue. Youth engagement was designed to empower youth as “future leaders” making a positive contribution to the development of their neighborhood as an alternative to participating in gang violence.

One powerful local system actor that GOAL did not seek to empower was gangs. Instead, GOAL worked to manage these actors and maintain neutrality, and GOAL never directly challenged gang agendas. Gangs were indirectly aware of GOAL’s activities through communication with community leaders who participated in the program, and the gangs implicitly accepted GOAL’s presence and activities, which they also benefited from as community members themselves. When the security risk posed by gangs was too high, GOAL would temporarily meet community members in a neutral area outside the neighborhood to progress the EWRS. Through careful management of security risks and close coordination and communication with community leaders, GOAL was able to ensure continuity of the program intervention with limited disruption of community involvement in a culture otherwise dominated by fear.

### Supporting and leveraging local assets

All communities have assets—even communities where conditions are relatively deprived. These include tangible assets—like infrastructure and equipment—and intangible assets—like knowledge and skills. Together, these tangible and intangible assets represent the input necessary for a self-sustaining and effective EWRS. GOAL worked to build upon existing assets, and temporarily filled in gaps where assets were lacking. This required overcoming three primary challenges. First, in contexts of informality and territorial gang violence, assets can suffer damages or not reach their full potential. Second, and partially as an extension of these conditions, Tegucigalpa and its neighborhoods often lack the necessary technical and material resources to help operationalize a comprehensive EWRS. The third major challenge in identifying and strengthening local assets was the pervasive lack of land tenure and legal ownership of assets. Where local people do not own local assets, they may be reluctant to invest their time and resources into improvements, though GOAL found that people were willing to invest in their homes and neighborhoods regardless of their legal ownership.

**FIGURE 7:**
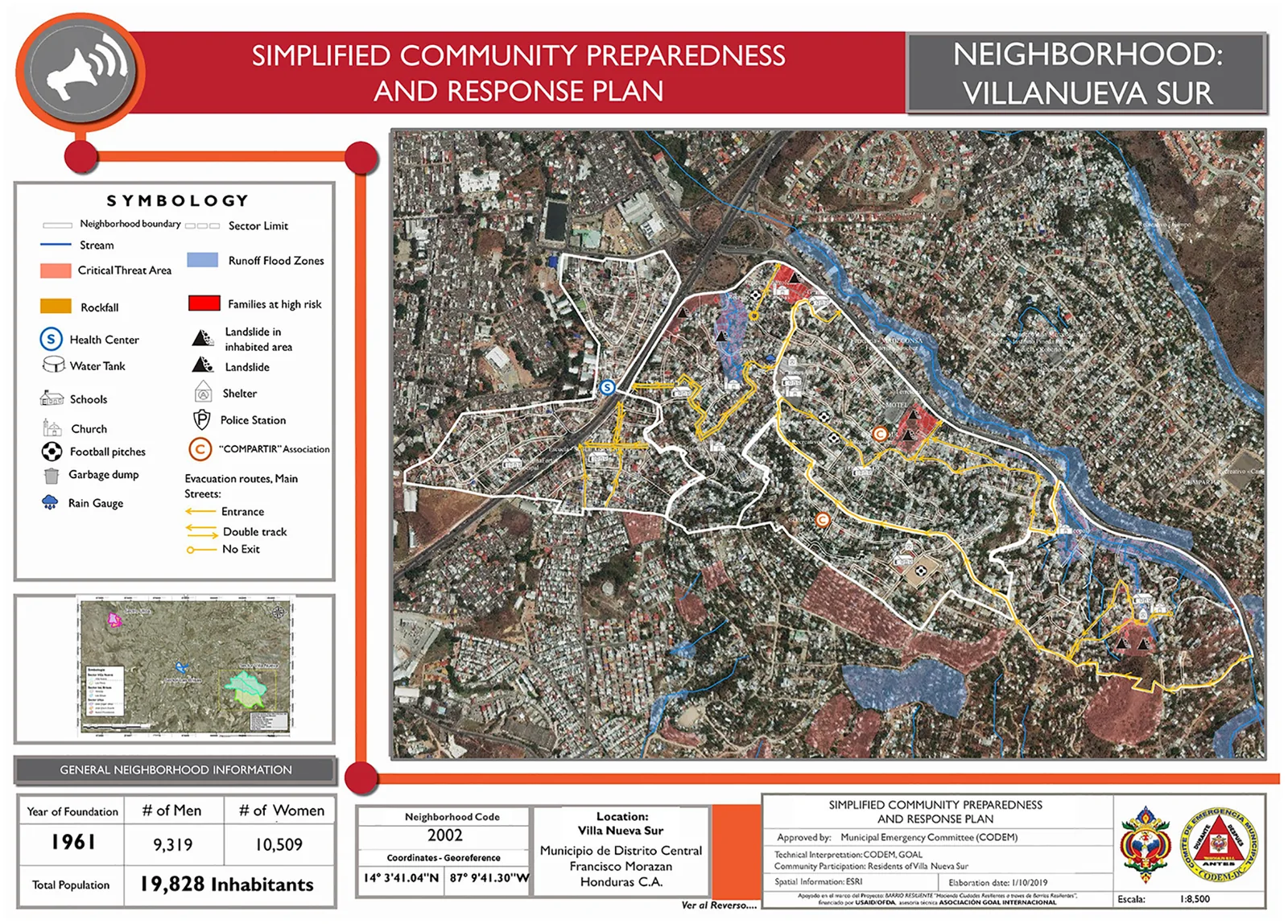
Simplified community preparedness and response plan including assets and hazards at the community level. Credit: GOAL, 2019.

**FIGURE 8:**
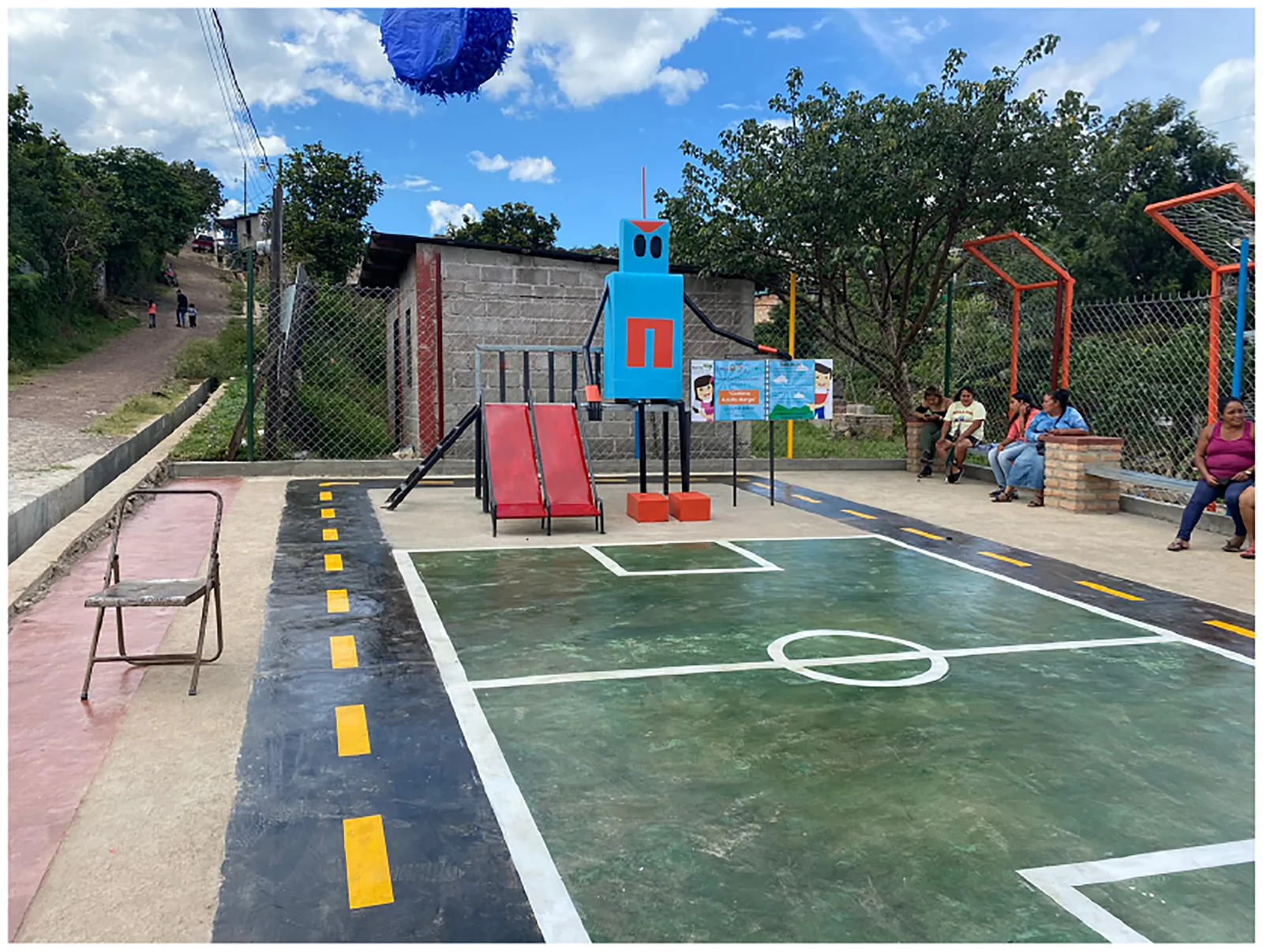
Playground constructed in a risk zone in an informal settlement. Credit: GOAL.

A participatory neighborhood census was at the heart of identifying and strengthening local assets. GOAL partnered with the municipality, community members, and Honduran university students to gather data on the socio-economic status of the community and available local assets. Going door to door, the census focused on identifying local assets that could be leveraged for the EWRS, including: infrastructure; public services; water and sanitation; health centers; educational centers and schools; community and recreational centers; religious centers and churches; small businesses, livelihoods and workspaces (like mechanic shops); supply storage spaces; and other public spaces (see Figure 7). These assets were digitized in a georeferenced inventory of local assets identifying those that (1) could contribute to preparation and response plans, (2) would benefit from improvement projects, and/or (3) posed high risks if they failed during an emergency. This mapping process was also critical to identify gaps in critical local assets and develop strategies together with local actors to address these gaps, such as limitations in capacities of available emergency evacuation centers, infrastructure for evacuation routes, or lack of healthcare facilities. Physical spaces in the community were identified as positive or potentially positive public spaces that could be used for community dialogues and activities to support improved social cohesion and conflict resolution needed for a functional EWRS. For example, GOAL identified vacant lots in high-risk areas and, with the support of communities and other partners, transformed them into playgrounds with the dual purpose of (1) preventing the construction of new homes in these spaces and (2) offering spaces for play and social cohesion for children, youth, and neighbors (see Figure 8).

**FIGURE 9:**
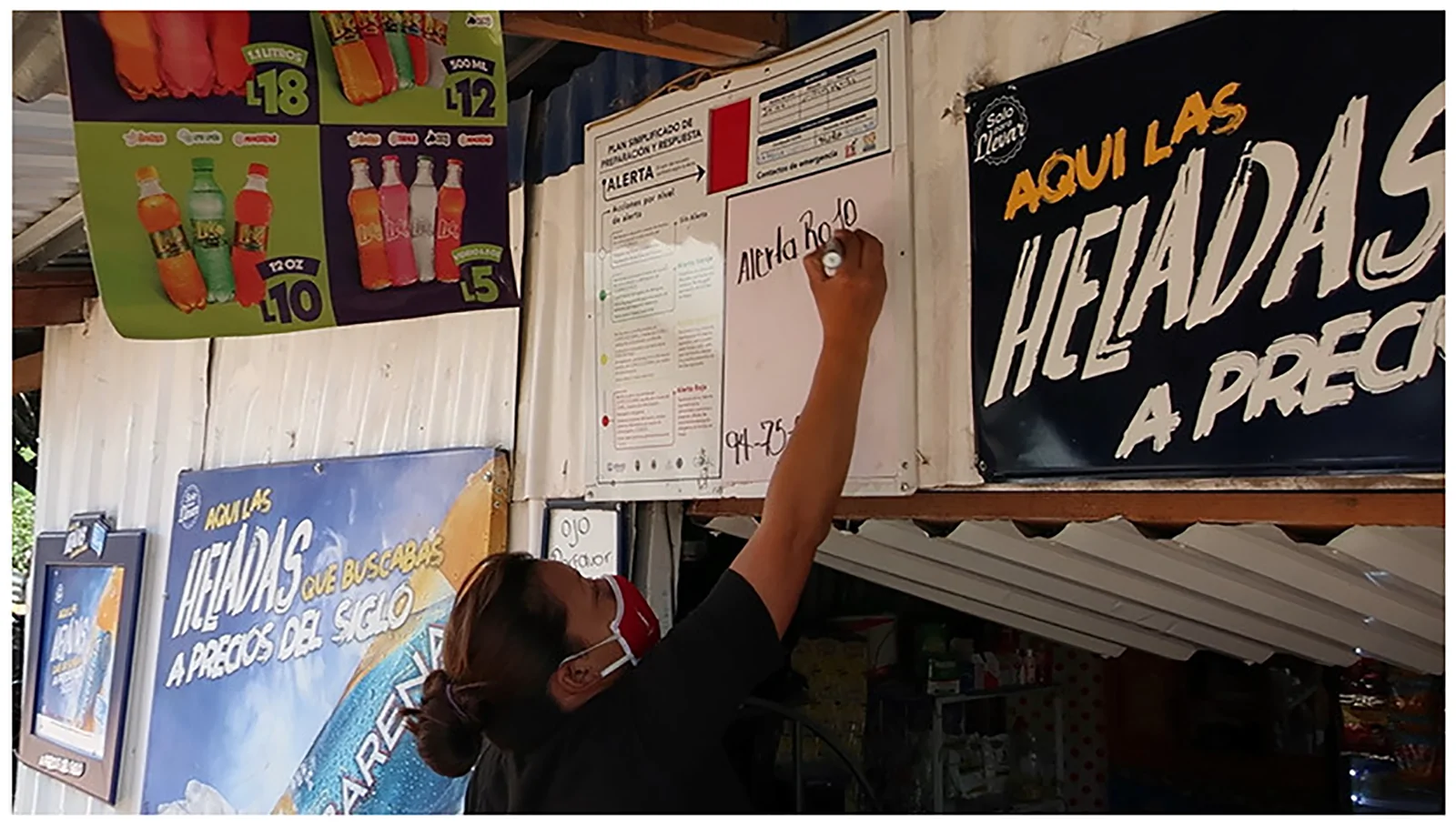
A *pulpería* owner in Tegucigalpa updates the EWRS alert level to Red Alert on a message board in her shop during an EWRS simulation exercise. Credit: GOAL, 2019.

By working with the local community, the participatory census led to the identification of several asset types that had not previously been viewed as critical. One was neighborhood stores, *pulperías,* and other micro, small, and medium enterprises owned and led by women and youth that provide critical goods and services to the urban informal settlements. These were highlighted as a useful asset for the operation of EWRS due to their position as community hubs and management and ownership by local women committed to the community (see Figure 9). A *pulpería-a-pulpería* (store-to-store) program was developed to facilitate networks and alliances among neighborhood stores to address common challenges like supply chain management, financial inclusion, and risk management. For example, GOAL’s programming improved their access to credit and savings, supported the establishment of networks of small and micro enterprises, and reduced their vulnerability to crime and other stressors. In doing so, the program sought to improve neighborhood access to services in places where formal service provision was limited, and help ensure service access continued during emergencies (McCaul and Nuñez, 2016). *Pulperías* were developed into key points for the EWRS. These stores became hubs for disseminating information and distributing emergency supplies—water, basic foods, nutrition supplements, cash, and communication equipment—and could be converted into response centers during and after emergencies. At the same time, by supporting and strengthening these local stores, GOAL also solidified trust and good will with the business owners.

Along with *pulperías*, monitoring capacities and equipment were also identified as critical local assets for the EWRS. Unlike *pulperías*, an asset already existing in the communities, in many cases monitoring capacities and equipment were lacking, so GOAL helped place monitoring equipment, such as rain gauges, wind speed sensors, river and reservoir level sensors, in key locations around Tegucigalpa. Similar to *pulperías*, community members were crucial to their functionality. For example, GOAL installed rain gauges in local volunteers’ homes to making monitoring rainfall levels easier: positioning this equipment in secure locations within the property of neighborhood residents made data collection both more convenient and safer than if it were in a public space exposed to insecurity, violence, and vandalism.

### Building cooperative systems of governance

Systems of governance refer to how EWRS resources are used, distributed, and managed. In Tegucigalpa, as elsewhere, governance is a hybrid mix of formal and informal structures. Formal governance refers to the control of resources in ways that are sanctioned officially by the state, while informal governance refers to the unwritten rules and understandings not codified within formal institutions or law. An effective EWRS depends on linking across formal and informal governance. However, informal settlements face the challenge of being excluded from formal city governance and services, and territorial gangs contribute to the complexity of the informal governance landscape. GOAL engaged with formal and informal institutions, such as community-based organizations, at multiple levels to help develop EWRS, promote partnerships in governance, and ensure the alignment of activities with global ambitions articulated, for example, in the Sendai Framework for Disaster Risk Reduction (UNISDR, 2015).

**FIGURE 10:**
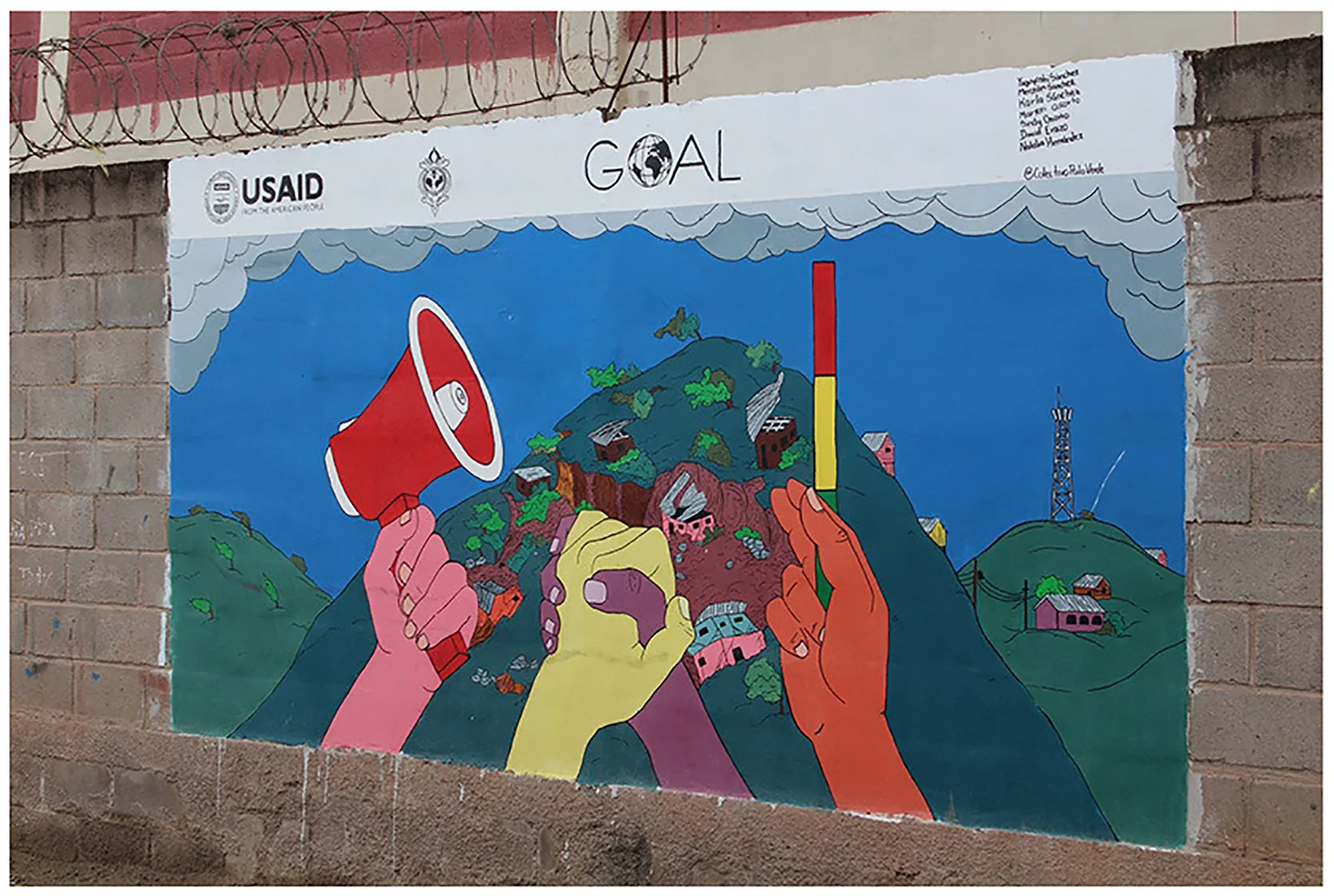
A mural showing landslide damage to a settlement (background) with a megaphone, gripping hands, and the colored alert levels from the EWRS (foreground). Credit: GOAL, 2022.

Some of these governance efforts centered on cooperative partnerships between local DRR units and those operating at the city and national institutional scales. For example, GOAL conducted training-of-trainer interventions so that the Municipal Emergency Committee (CODEM) could train local volunteers. The CODEM oversees the Local Emergency Committees (CODEL), which are community-led groups made up of 10–20 volunteers who are often community leaders that respond to disasters. CODEL leads monitoring and communication, coordination of first responders, shelter and humanitarian relief, and analysis of damages and community needs, and each CODEL is also responsible for developing and implementing tailored community risk management plans for neighborhoods in partnership with their communities. CODEM and CODEL collaborate during emergencies, bridging the gap between informality and formality and working toward a coordinated response (see Figure 10).

GOAL worked to increase the cooperative governance capacities of municipal authorities and community members. In part this was done by building community capacity to work with “experts” at the city and national levels and in other part by encouraging formal institutions to treat community structures as legitimate partners in EWRS. Local legitimacy and expertise was sometimes questioned, for example when political or community leadership changed. Such changes in personnel often required trust-building between institutions to begin from scratch—a task which could not be diminished or taken for granted. In addition to trust-building, it was essential to maintain clear roles and a well-defined distribution of authority within collaborative governance structures, including to specify that GOAL’s role was as a temporary facilitator and not the leader. This explicit statement of local ownership was important for situating the EWRS as a local system with local actors as permanent partners rather than an initiative to be absorbed by municipal authorities. When appropriate, GOAL also supported the establishment of formal agreements with neighborhood committees and other key governance structures outlining decision-making and dispute-resolution processes.

As part of the coproduction process, GOAL worked to understand the long-term goals of communities, which included the desire to maintain their own power and authority rather than be replaced by a formal city governance structure far removed from their everyday concerns and lives. Community leaders involved in the EWRS worried that due to their social and legal status in informal settlements, they would eventually become excluded from decision-making processes. In response, GOAL provided community leaders with leadership training focusing on relevant topics such as effective communication, conflict resolution, and collaborative planning in complex governance contexts, including related to informality and territorial gang violence. GOAL paradoxically found that the potential for social cohesion and cooperative action was even greater in high-risk neighborhoods exposed to hazards and gang violence in comparison with middle-income neighborhoods. The public desire to improve their neighborhoods and gain access to basic services motivated them to take collective action for the benefit of their community. While it is not a simple or easy task to mobilize people and resources, community leaders learned strategies to bring people together and identify and work toward common goals even in fraught contexts. Community leaders held bimonthly meetings to communicate actions to communities with the objective of reinforcing their leadership, promoting accountability necessary for good governance, and assuaging and addressing community concerns.

### Engaging with dynamic local systems

Local systems are dynamic, changing as the city evolves and grows. EWRS must adapt to these changes to remain responsive to the evolving risk landscape. Dynamism is even more prevalent in urban informal settlements, which are not bound by the same degree of policy constraints and, in the context of Tegucigalpa, are characterized by high levels of in- and out-migration. Territorial gang violence also contributes to sometimes rapid and unexpected changes in security as well as the overall functioning of the EWRS—for example, during spikes in violence, outside vendors may temporarily suspend services that are necessary for disaster mitigation, preparation, and response.

GOAL positioned itself as a facilitator to ensure that evolving needs remained met and that capacities continued to grow, while reinforcing local ownership of these systems. GOAL became familiar with the target neighborhoods and local actors through regular vulnerability, needs, and asset assessments to track changes over time. Local system mapping and analysis using tools such as R4S that GOAL developed (GOAL, 2019) brought local system dynamics into focus and how they might be impacted by risk scenarios. These participative exercises facilitated shared understandings of neighborhoods, local actors, and their priorities as well as the threats they faced, such as sometimes fluid territorial gang control and violence. GOAL’s long-term presence was beneficial, as it allowed for familiarity and relationships with dynamic and changing communities to grow throughout the entire course of programming.

GOAL supported elements of consistency and stability for local systems within this dynamism. Knowledge and skills were not centralized in these communities, which brought challenges but also ensured necessary redundancy in the context of dynamic change. This decentralized stability was exploited to build sustainability into EWRS efforts; interventions targeted stable nodes or demographic networks themselves—such as women business leaders—that could hold knowledge and maintain functionality of the system regardless of residency churn at the individual level. GOAL reinforced this stability through conducting training-of-trainer workshops covering topics like technical capacities and understanding local risks from inclusive perspectives. Establishing such mechanisms to institutionalize the transfer of knowledge and skills promoted the self-sustainability of the EWRS into the future.

**FIGURE 11:**
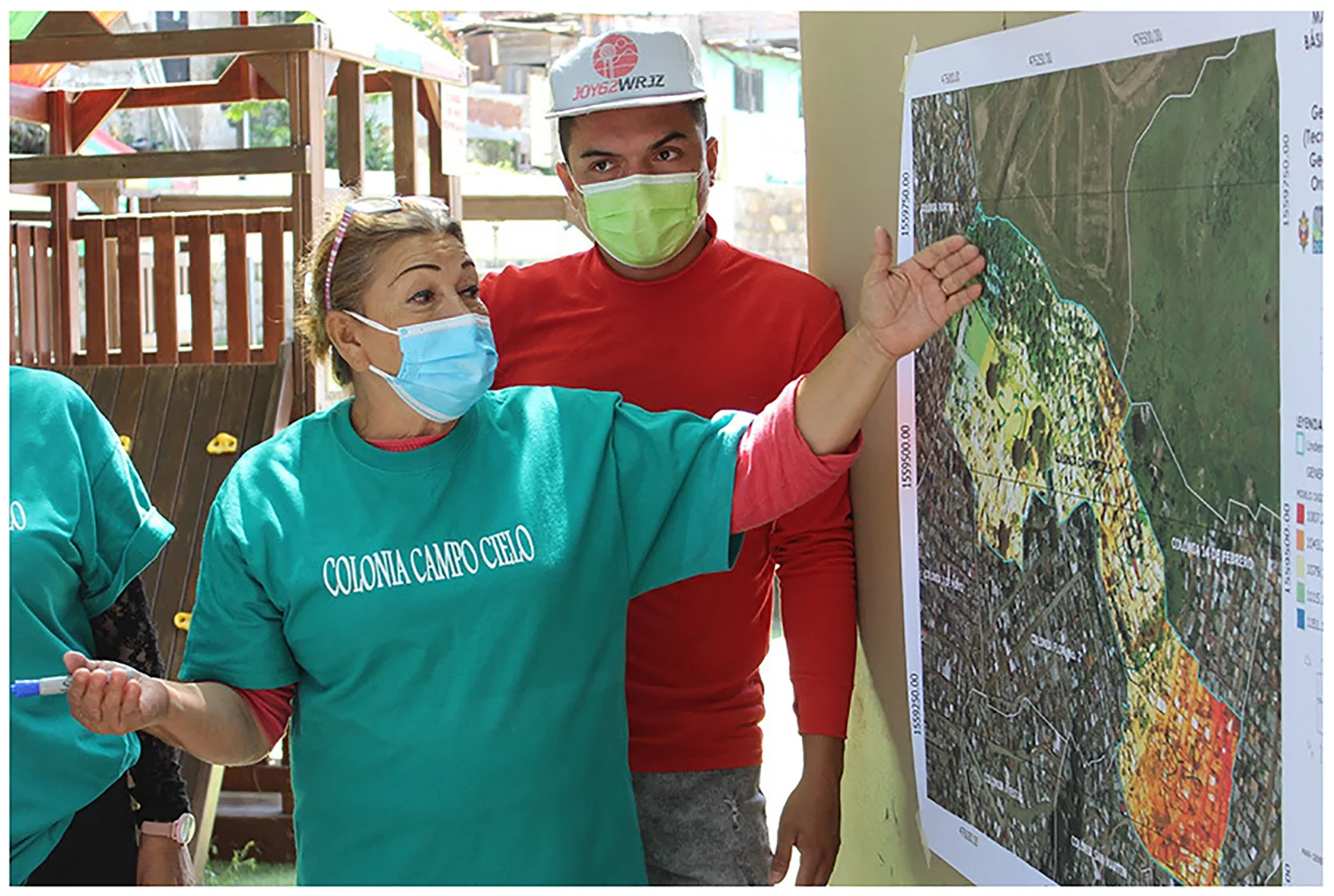
GOAL supported risk awareness in neighborhoods through collaborative processes, like the public group discussion of the hazard map shown here in July 2021 in the Campo Cielo community in Tegucigalpa. Credit: GOAL, 2021.

GOAL developed a dynamic communication strategy at the start of its engagement to build top-down, bottom-up, and neighborhood-to-neighborhood communication and connection amongst authorities, community leaders, neighboring communities, and other stakeholders. This strategy included face-to-face engagements, group engagements through public message boards and loudspeakers, and leveraging communication platforms including text messages, Facebook, and WhatsApp groups. GOAL and key local actors designed communication strategies together, focusing on both explaining EWRS, including realistic expectations about what EWRS can and cannot accomplish, and providing information about their rights in the context of informality (see Figure 11). These mechanisms also facilitated ongoing community feedback in real time, which informed when and how changes and adaptations needed to be made.

## Discussion and policy recommendations

This article examined the coproduction of EWRS in nine urban informal settlements controlled by territorial gangs in Tegucigalpa, Honduras. Informality and territorial gang violence reflected and created high vulnerability extending to disaster risks, and they also challenged best practices for bridging the gaps between knowledge and action in EWRS. The results demonstrated how a local systems approach enabled GOAL to adapt coproduction to conditions of informality and territorial gang violence, which they pursued through four cross-cutting strategies: (1) identifying and empowering key local systems actors; (2) supporting and leveraging local assets; (3) building cooperative systems of governance; and (4) engaging with dynamic local systems. These strategies allowed GOAL to maintain access to and engage with communities affected by territorial gang violence and support cooperative governance structures amidst informality.

GOAL’s work highlights the necessity to engage with EWRS as a social process rather than treating it as an apolitical technical exercise. A local systems approach may be particularly effective in coproducing EWRS in challenging contexts affected by informality and territorial gang violence, as it explicitly pursues solutions rooted in complex interactions. Focusing on interactions rather than standalone components allowed the EWRS to tailor and target interventions to the areas which can most effectively pressure system improvements and transformations. GOAL never approached standalone actors to work on components of the EWRS but worked through consortia, and a side effect was creating more powerful and inclusive coalitions for the collective good. People disproportionately victimized by violence, including women and youth, were empowered to occupy leadership positions and co-develop a vision for change, but focusing interventions at the city level redistributed the burden for this change. This approach enabled women and youth to have important roles in coproducing the EWRS that could be sustained over time without depleting their capacities within other essential local systems. This local systems approach may provide implementing agencies with a lens to support local capacity for EWRS and assist in alleviating the systemic drivers of their vulnerability and exclusion.

GOAL helped persuade those creating and benefiting from the status quo of vulnerability to find shared interests and see the benefits of championing inclusive EWRS. Transformative DRR agendas are not uniformly positive and may be used as an extension of broader political agendas that can threaten people (Peters and Kelman, 2020) and may even oppose the very existence of informal settlements (Morin et al., 2016). This reaffirms that “there are competing visions for DRR in conflict situations” (Siddiqi et al., 2019, p.8), and those who intervene become part of the conflict, for better or worse through aligning with or pursuing specific agendas. Rather than seeing this as an excuse to implement anemic support or not intervene at all, GOAL worked to facilitate rather than drive a vulnerability reduction agenda by co-creating a vision for change with local systems stakeholders.

Developing a genuine EWRS calls into question how to engage complex governance arrangements (including multilayered relationships between the state and formal urban governance, territorial gangs, and other community groups) and neighborhoods under their control. On the one hand, local EWRS must be connected to broader EWRS operating at city-wide and national levels in order to be effective. Thus, the government with formal authority and mandate must be involved and persuaded to integrate informal settlements into centralized services. On the other hand, territorial gangs must be included to some degree in activities in areas which they control, in part because such activities “will be subject to discussion or authorization by the gang, whether one is aware of it or not” (Bangerter, 2010, p. 400). Beyond the reality of needing to gain tolerance or be invited to conduct activities, territorial gangs may serve prosocial roles, albeit limited, in these neighborhoods, and engaging in dialogue with gang leaders and members can provide security and build mutual trust and understanding (Bangerter, 2010) that underlie participatory processes.

Working with key local systems actors in FCAC can thus come with a price, especially where the line between benevolent and predatory actors is blurred. GOAL avoided liaising directly with territorial gangs; gangs implicitly accepted GOAL’s presence and maintained an awareness of—without direct involvement in—EWRS activities. Despite this, GOAL at times had to severely limit programming due to physical security risks. In other urban FCAC where territorial gangs are major service providers, this avoidance strategy may not be possible. Territorial gangs can be a source of social and economic opportunities for associated family members and the community at large (Bangerter, 2010), and they can provide basic services—including DRR—in FCAC (Walch, 2018). Territorial gangs, including in informal settlements in Tegucigalpa, are invested in making their communities safer and more livable for themselves and their families as well as to build their own authority, but territorial gangs also paradoxically thrive on vulnerability. Organizations working even indirectly with gangs may confer upon these predatory entities an element of legitimacy, strengthen their authority, and allow them to claim some delivery on a social contract with the community (Doyle, 2021). This may engender and reinforce vulnerability and, especially in contexts of informality, undermine the perception of the need to extend formal city services to all settlements. In this sense, an effective EWRS dependent on territorial gang involvement could resolve some elements of fragility while cementing others.

GOAL’s local systems approach helped them support EWRS for a relatively low cost, especially when considering the high human and financial cost of disasters. A local systems approach may be more economical than pervasively fragmented approaches to disaster management particularly in informal settlements. At the same time, not all vulnerable communities across Tegucigalpa could be supported simultaneously and with the same degree of coproduction. The assets developed or delivered in these communities such as EWRS machinery or the education, health, and work centers stand in contrast to their deficit in other communities. Interventions can thus carry the unintentional consequence of changing the balance of relative vulnerability within the system by reducing vulnerability in one area and leaving them to stagnate somewhere else; DRR often never reaches the most vulnerable communities in FCAC, instead focusing on relatively more stable alternative locations (Peters, 2021). Especially in urban FCAC, this can lead to violence concentrating in relatively more vulnerable neighborhoods and/or play into tensions and further conflict between rival neighborhoods. On the other hand, research has also found that marginalized communities without formal ownership and rights may be reluctant to engage in DRR that improves their neighborhoods out of fear that they will be evicted (Peters, 2022). This reaffirms the necessity for transparency, trust, and accountability in EWRS to monitor and redress unintended consequences, and to continuously pursue improved inclusion. These mechanisms can be hard to cultivate in urban FCAC and in this case required continuous facilitation by GOAL.

The local systems approach GOAL developed to coproduce EWRS shares similarities with area-based programming, which refers to organizing aid around the multisector needs of a defined community or geography rather than around the technical expertise of aid agencies (Konyndyk et al., 2020; Haider, 2021). Often implemented in FCAC, area-based programming seeks to create synergies in programming (Fraser, 2011), but it can perversely deepen inequities and conflict and lead to shortfalls in meaningful community participation (Haider, 2021). The local systems approach emphasized local capacity-building and relationship-building as two of the centerpieces of GOAL’s programming, rather than seeing people living in informal settlements as solely vulnerable and passive recipients of aid. The local systems approach also takes area-based programming one step further in acknowledging the interactions between institutional scales that catalyze and constrain transformations at the heart of DRR. In doing so, a local systems approach targets both the area and the wider systems that impact that area, bringing to light where focused interventions can yield disproportionately positive impacts. This local systems approach thus can reconcile and mitigate a noted risk of area-based programming, which can lead to isolated interventions that are not integrated into wider municipal plans and systems (Sanderson, 2017).

This study has several implications for policy and practice for EWRS in urban FCAC:

1. Donors and practitioners must find ways to foster collaboration not only in informal settlements but also across the city. EWRS are a last line of defense for vulnerable people exposed to hazards so that they can evacuate and take preparedness actions with adequate time. However, even the earliest actions are not early enough without engaging with and reducing vulnerability, including building cooperative forms of informal and formal governance. An emphasis on climate change and technology-based solutions alone distracts from the hard work necessary to address social and political factors responsible for the exclusion of people living in informal settlements from EWRS and DRR more broadly.
2. EWRS cannot be designed and implemented outside of local participation, demonstrating the need for first-mile and bottom-up approaches to the creation of inclusive knowledge and action (Kelman and Glantz, 2014). Investments must be made to understand how to support critical local systems including in urban FCAC where they are needed to reduce disproportionately high disaster vulnerability but are undermined by high levels of violence, instability, and dynamism. Given rapid rates of urbanization and increasing populations of urban informal settlements (UN Habitat, 2018), learning in these contexts should be the new “conventional” starting point for developing transferable coproduction strategies for EWRS.
3. Longer-term and more dynamic and flexible approaches to funding might be required in urban FCAC, including those that better apply multi-hazard and compound risk information (Kruczkiewicz et al., 2021). There is evidence that multi-year flexible funding and flexible adaptive partnerships can maximize the benefits from interventions (IRC, 2020) particularly with transformative DRR ambitions coproduced and owned by communities. Relatedly, funders should invest in capacity- and relationship-building among local and international NGOs in order to develop coordinated long-term strategies.

This study has limitations, and there are significant areas of further attention and research to improve the development and deployment of EWRS in urban FCAC. The local systems approach to coproducing EWRS presented in this manuscript is designed to be scalable and transferable, but further research must be conducted to assess how well-these strategies can be adapted to different contexts and different institutional scales. GOAL is currently replicating their approach in three other cities in Northern Honduras (Villanueva, El Progreso, and San Pedro Sula), which are at high risk of flooding and affected by high levels of gang violence and social exclusion, as well as in two cities in Colombia (Bucaramanga and Barranquilla), which are at high risk of flooding and landslides in the case of Bucaramanga and runoff flooding and social violence against LGBTQI people and migrants in the case of Barranquilla. These cases make future comparative research possible. This research was coproduced with GOAL, which impacts the positionality of the manuscript. It would be valuable to engage community members and other stakeholders to bring more perspectives into the research and strengthen the validity of the findings. As more extreme climate hazards affect expanding informal settlements, it will remain to be seen the extent to which coproduced EWRS stand up as a line of defense for these communities.

## Conclusion

Anticipatory DRR—including in urban informal settlements controlled by territorial gangs—is a social and political process that coproduction can facilitate, enable, and shape. Even the technical aspects of an EWRS monitoring system—like satellite observations, forecast modeling, and ground-based data—require all-stakeholder engagement and are far from being a technocratic endeavor. Yet, because stakeholders ranging from state and local governments, informal governance, and affected people are not on an even playing field, EWRS remains a social and political process that may benefit from facilitation by outside agencies like GOAL. Through long-term partnerships, they can co-create change leading to vulnerability reduction at the core of disaster risk. In urban FCAC, this requires navigating conditions of fragility and conflict which both reflect and create disaster risks—and challenge humanitarian and development initiatives.

Despite the challenges and constraints, GOAL’s work is also a picture of possibility. Disaster risks are politically and socially generated, which means that they can be addressed through appropriate human actions that are within our reach. Our research highlights an innovative local systems approach to coproduction that fosters long-term engagement and nurtures a culture of preparedness to achieve a real reduction of risks. This multisectoral approach to coproducing inclusive EWRS grounded in communities affected by informality, violence, and disaster risks in Tegucigalpa has the potential to be scaled to other urban FCAC and contribute to the global ambition of leaving no one behind in DRR.

## Figures and Tables

**TABLE 1:** The EWRS incorporates four alert levels, increasing in severity from No Alert to Red Alert, with corresponding actions for different stakeholders at the community level, institutional level, and first responders.

Alert level	Community actions	Local emergency committee (CODEL) actions	Siren alarm (in addition to other alert communications)
No alert	Monitor communications from media and CODEL Maintain 72-h emergency kits Engage in mitigation and preparation activities	Transmit risk information to at-risk populations Confirm evacuation routes Promote personal harm-reduction activities	Siren does not sound
Green alert	Monitor for alerts and hazard conditions relating to flooding or landslides Support the distribution of emergency supplies and equipment	Initiate communication of possible voluntary evacuation advisory Prepare emergency evacuation shelters Notify the most vulnerable community members	Siren sounds 3 times
Yellow alert	Continue to monitor for alerts and changing conditions Begin voluntary evacuations for the most vulnerable households and at-risk zones	Transmit voluntary evacuation advisory Activate emergency evacuation shelters Assist community members who require assistance	Siren sounds 5 times
Red alert	Mandatory evacuation from high-risk zones Monitor alerts from authorities	Transmit mandatory evacuation orders Evacuate CODEL members themselves	Siren sounds continuously

Local Emergency Committees (CODEL) are community-led, volunteer groups that respond to disasters, and discussed in Section 4.3 below.

**TABLE 2:** External evaluation of GOAL’s contribution to DRR and social cohesion through their EWRS programming, adapted from Florida International University (2018).

DRR index	Score
Community has members trained in DRR	71.8
*Project contributed to it (agree and strongly agree)*	59.1
Community has motivated members who support DRR	94.9
*Project contributed to it (agree and strongly agree)*	79.5
Community has a functional EWS including drills	64.9
*Project contributed to it (agree and strongly agree)*	50
Community involved in the emergency plan implementation	62.2
*Project contributed to it (agree and strongly agree)*	45.5
Community involved in maintenance of projects’ physical works	81.4
*Project contributed to it (agree and strongly agree)*	70.5
Social cohesion index	Score
Strong sense of belonging to this neighborhood	100
*Project contributed to it (agree and strongly agree)*	90.9
Living here gives you a sense of community	97.7
*Project contributed to it (agree and strongly agree)*	86.4
Willingness to work together to improve your neighborhood	100
*Project contributed to it (agree and strongly agree)*	88.6
Neighbors would help each other during an emergency	95.5
*Project contributed to it (agree and strongly agree)*	88.6

## Data Availability

The original contributions presented in the study are included in the article/supplementary material, further inquiries can be directed to the corresponding author/s.
